# Emulsification of Surfactant on Oil Droplets by Molecular Dynamics Simulation

**DOI:** 10.3390/molecules25133008

**Published:** 2020-06-30

**Authors:** Yaoshuang Cheng, Shiling Yuan

**Affiliations:** Key Lab of Colloid and Interface Chemistry, Shandong University, Jinan 250199, China; yaoshuangcheng@mail.sdu.edu.cn

**Keywords:** asphaltene, SDSn, molecular dynamics, heavy oil, emulsification

## Abstract

Heavy oil in crude oil flooding is extremely difficult to extract due to its high viscosity and poor fluidity. In this paper, molecular dynamics simulation was used to study the emulsification behavior of sodium dodecyl sulfonate (SDSn) micelles on heavy oil droplets composed of asphaltenes (ASP) at the molecular level. Some analyzed techniques were used including root mean square displacement, hydrophile-hydrophobic area of an oil droplet, potential of mean force, and the number of hydrogen bonds between oil droplet and water phase. The simulated results showed that the asphaltene with carboxylate groups significantly enhances the hydration layer on the surface of oil droplets, and SDSn molecules can change the strength of the hydration layer around the surface of the oil droplets. The water bridge structure between both polar heads of the surfactant was commonly formed around the hydration layer of the emulsified oil droplet. During the emulsification of heavy oil, the ratio of hydrophilic hydrophobic surface area around an oil droplet is essential. Molecular dynamics method can be considered as a helpful tool for experimental techniques at the molecular level.

## 1. Introduction

Asphaltenes are a class of polycyclic aromatic hydrocarbon compounds in crude oil, insoluble in paraffin medium, such as n-heptane, easily soluble in aromatic crude oil and organic solvent such as o-xylene [1,2]. The natural asphaltenes in heavy oil tend to accumulate in the crevices of rock and they can also be blocked in wellbore tubing and other equipment [3,4], which greatly reduces the oil recovery rate [5] and increases the cost of oil displacement in the oil industry [6,7,8]. In heavy crude oil, there are many types of asphaltenes [9]. Among them, asphaltenes containing carboxylate are amphoteric, easy to stay at the oil-water interface, and have strong surface activity [10,11], which plays a key role in the emulsification of heavy oil. In recent years, a lot of researches have been conducted on the aggregation of asphaltenes [12] and the stability of oil-water emulsions [13] has been discussed, and various techniques [14,15,16,17,18] have been used to analyze the effects of emulsifiers [19] on asphaltene molecules. For example, micropipette technology [20] was used to study the formation and destruction of the asphaltene molecular membrane at the water/oil interface [21,22,23,24]. Atomic force microscope (AFM) was used to observe the morphology of asphaltene film on the glass or silicon surface [25,26].

However, experimental research commonly ignored the behavior of surfactant emulsifying heavy oil at the molecular level. Computer simulation techniques such as molecular dynamics (MD) and Monte Carlo (MC) methods are effective supplements to the experimental research at the molecular level. These simulations can well describe the emulsifying behavior of surfactants at the oil/water interface, and reveal the microscopic properties of asphaltenes [27]. The simulated results showed that the main mechanism of surfactants enhanced oil recovery [28] is that surfactants can reduce the oil/water interfacial tension and change the interfacial property of heavy oil and water phase [29]. The simulation results of Tang et al. [30] indicated a three-stage process of surfactant flooding driven oil-detachment, including the initial formation of surfactant micelles and delivery, the disintegration-spread and migration of surfactant molecules on the oil aggregate. Bhattacharjee et al. studied the aggregates of asphaltenes in water/organic solvent systems [31]. They found that the model molecules tend to form aggregates in pure solvents, and the interfacial activity of asphaltenes may be related to the heteroatom properties, but not to polycyclic aromatic hydrocarbons. Gao et al. [11] used C5Pe containing carboxylic acid as the asphaltene model molecule. They found that the C5Pe molecules with anionic groups can be paralleled to the oil-water interface. Su et al. [32] studied the emulsification and viscosity reduction heavy oil after surfactant molecules were added, and the simulated results showed that the reduced viscosity of heavy oil is related with the hydrophilicity and emulsification performance of oil/water surface. However, the emulsification mechanism of surfactant micelle in oil/water emulsion still could be discussed at the molecular level.

In this work, molecular dynamics (MD) simulation is used to study the adsorption structure of sodium dodecyl sulfonate (SDSn) micelle on the surface of a heavy oil droplet, and the effect of asphaltenes was discussed on the emulsification of heavy oil.

## 2. Results

### 2.1. Aggregation Structure

Root mean square displacement (RMSD) represents the position deviation of the atomic position coordinates from the initial moment. The change of the distance of a component relative to its initial position, and the motion of the substance in the system are observed from the RMSD [33], and then the thermodynamic equilibrium of the system was judged by it. The calculation formula (1) is as follows:

(1) $$\mathrm{RMSD}=\sqrt{\frac{1}{N}\overset{N}{\underset{i=1}{\sum }}{\langle \left|r_{i}\left(t\right)-r_{i}\left(0\right)\right|\rangle }^{2}}$$

where N is the number of atoms, $r_{i}\left(t\right)$ is the position coordinate of the i-th atom at time  t, and $r_{i}\left(0\right)$ is the initial position coordinate of the i-th atom.

**Figure 1:**
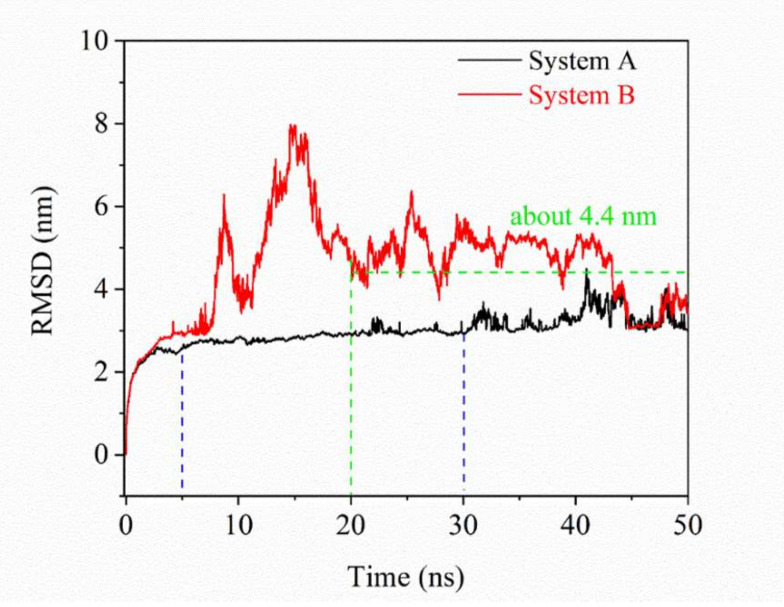
RMSD of oil droplets in water.

**Figure 2:**
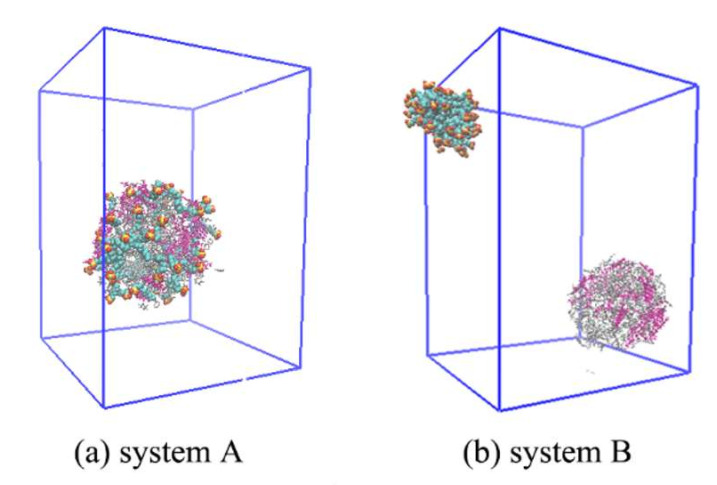
The configuration diagram at 50 ns in (**a**) system A (**b**) system B. SDSn are displayed in blue, yellow, and red spheres, ASP are displayed in rods and marked with rose red, and other heavy oil molecules are marked in gray. To be shown clearly, sodium ions and water molecules are removed.

The RMSD with only oil droplets is shown in Figure 1. Meanwhile, the RMSD of the surfactants is also shown in Figure S1. In the first 5 ns, the RMSD of oil droplets both in system A and B increase rapidly. This is because the systems relaxed rapidly from the spherical oil droplet at the beginning of the simulation. In system A, the RMSD did not change much within 5–30 ns, indicating that the oil droplet structure is stable and difficult to change. We noted that the RMSD of the oil droplet molecules in system B increase significantly within 5–20 ns. This is because the structure of the oil droplets changed greatly. After 20 ns, there are still significant fluctuations, indicating that the oil droplet in system B is relatively unstable. The RMSD after 20 ns was calculated, and the average is about 4.8 nm. From the RMSD of oil droplets in two systems, it can be concluded that the carboxylate asphaltene in system A can enhance the diffusion of SDSn micelle and make the system more stable. During the simulation period, when the system reached the equilibrium stage at 50 ns in Figure 2, the micelles adsorbed in the oil droplets of system A, but not in system B. With the lengthening of the simulation time, both adsorption and emulsification occur (Figure S2), but the system A adsorbs faster, which proves that the acidified asphaltenes are very important for forming emulsified oil droplets. This indicates that the acidified carboxylate group of the asphaltenes is crucial to the formation of emulsified oil droplets.

### 2.2. Emulsification Process

**Figure 3:**
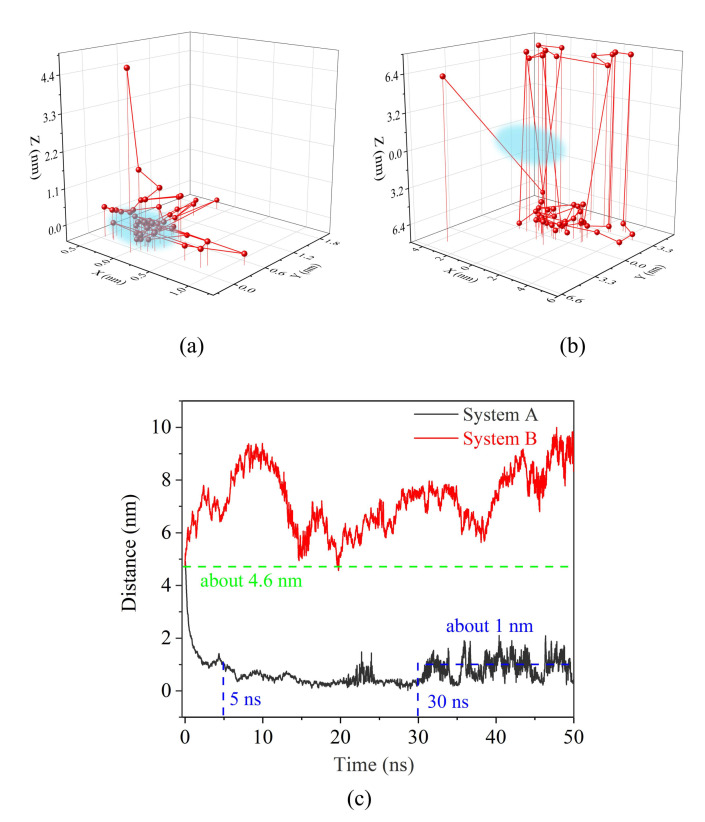
The distance as a function of time between the centroid of the SDSn micelle and the centroid of the oil droplet in (**a**) (**b**) direction (x, y, z) (The blue area is the range of motion when they gather) (**c**) the straight-line distance.

To gain more insights into the relative position between SDSn molecules and oil droplets, and to determine whether SDSn molecules are emulsified with oil droplets, the distance as a function of time about the centroid of them was quantified, as shown in Figure 3. For system A, the distance in Figure 3a decreases sharply from 4.2 nm to 0 nm in the Z direction, and finally fluctuates at 0 nm. Meanwhile, there is a slight fluctuation around 0 nm after gradually decrease in the X and Y directions. This shows that the micelle molecules are quickly adsorbed on the oil droplet during the simulation, and the center of mass of the SDSn and the oil droplet are approximately coincident with the oil droplets during the aggregation process. In Figure 3c, the linear distance between the SDSn molecular centroid and the oil droplet centroid decreases rapidly during 0–5 ns in system A, indicating that adsorption occurs quickly. Within 5–30 ns, the linear distance drops slowly and tends to 0 nm. But for the B system the distance between the two centroids (x, y, z) in Figure 3b shows a periodic change in the Z direction up and down and is away from the coordinate (0, 0, 0) where the centroids of the two coincide. As shown in Figure 3c, there is a significant fluctuation in the straight line distance which is almost always much longer than the initial distance of 4.6 nm. This indicates that the adsorption of the micelle in system B has never occurred. So next we focus on system A to study the mechanism of the emulsified oil droplet.

**Figure 4:**
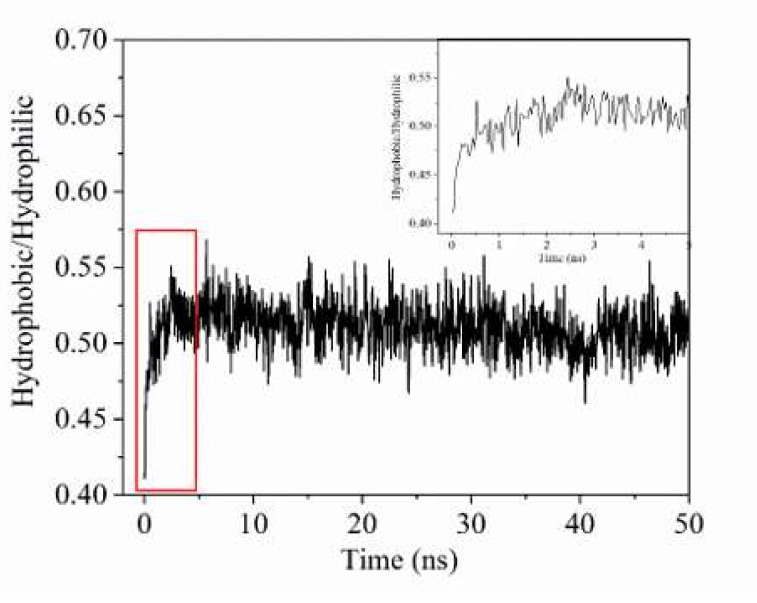
The ratio of hydrophilic/hydrophobic area of SDSn and oil droplet in system A and the inset is the ratio from 0 to 5 ns.

Solvent accessible surface area is the surface area where the spherical probe rolls around the periphery of the target molecule, and the contact area generated by the aggregation inside the target molecule is not included. The hydrophilic and hydrophobic properties of the model molecules can be characterized by the hydrophilic solvent accessible surface area and the hydrophobic solvent accessible surface area. To study the properties of the oil-water interface during the adsorption of micelle by the oil droplet, solvent accessible surface area (SASA) of the oil droplet and SDSn as a function of time was calculated. Figure 4 shows the ratio of hydrophilic/hydrophobic area of the SDSn and oil droplet in system A. From 0 to 5 ns, the hydrophilic/hydrophobic surface area of oil droplet and SDSn molecules in system A increase rapidly from 0.36 to 0.52, and then fluctuate slightly, indicating that they in system A have been fused during this period.

**Figure 5:**
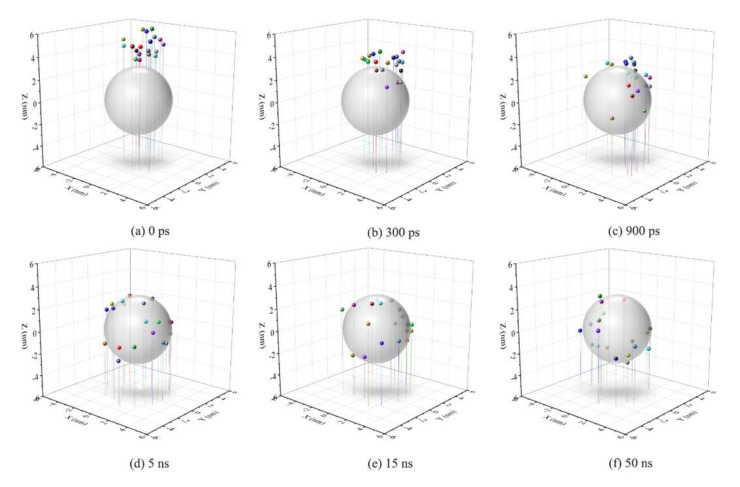
The distribution of SDSn polar head coordinates over time. Oil droplets were represented with gray spheres, and 20 SDSn molecules are randomly selected from all SDSn.

**Figure 6:**
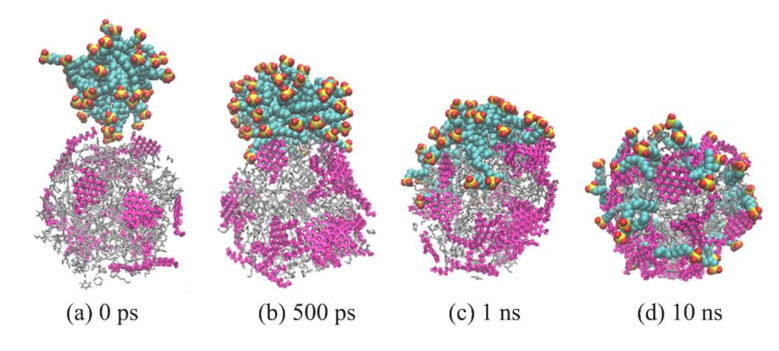
Snapshots of system A without sodium ions and water at different time. (**a**) 0 ps, (**b**) 500 ps, (**c**) 1 ns, (**d**) 10 ns. SDSn were represented with the spherical drawing method, and heavy oil molecules were represented with bond drawing method, color identification: rose-red, asphaltene molecules, gray, other oil molecules.

Figure 5 shows the distribution of SDSn polar head coordinates with time evolution, and Figure 6 shows the configuration changes of the SDSn molecules and oil droplets in the simulation of system A. Combining the two figures, at the initial moment, the micelle and oil droplet were randomly placed without contact (Figure 5a and Figure 8a). In a short time, due to the hydrophilic effect of the polar heads of surfactant molecules and asphaltene molecules, they formed hydrogen bonds with water molecules to form the water bridge structure. After that, the tail chains of SDSn were separated from the micelle, and their alkyl chains were close to the hydrophobic molecules in the oil droplets, and the polarity head of SDSn molecules was close to the amphiphilic asphaltenes on the oil droplet surface which SDSn molecules reach (Figure 5a–d). Subsequently, under the hydrophilic effect of the polar heads of the SDSn and the hydrophobic effect of the alkyl chains, the polar heads of SDSn are oriented towards the solvent water, and the alkyl tail chains are inserted into the oil droplets (Figure 6b). With the extension of the simulation time, due to the hydrophobic interaction between the hydrophilic groups, amphiphilic molecules such as SDSn and asphaltenes are almost evenly distributed on the surface of the oil droplet (Figure 5e,f). So that’s the emulsification (Figure 6a–6d). Therefore, the addition of SDSn changes the hydrophilic/lipophilic surface area of the oil droplet and increases the hydrophilic surface area of the oil droplet.

**Figure 7:**
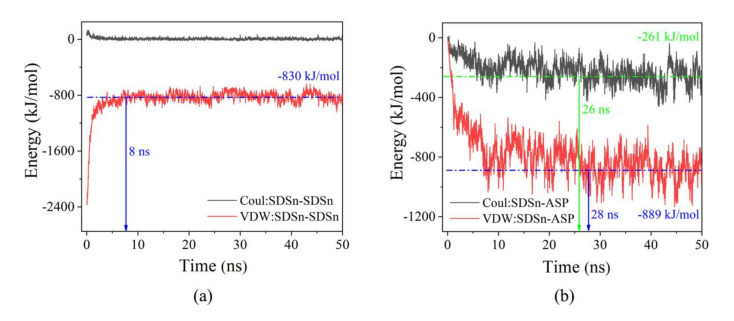
Interaction energy of SDSn molecules with (**a**) SDSn and (**b**) ASP.

The adsorption of micelle molecules and oil droplets is mainly caused by non-bonded interactions, which include electrostatic interaction (Coulombic) and Van der Waals interaction (Lennard Jones). The difference between systems A and B is only whether they carry carboxylate groups on asphaltenes. Therefore, we focus on the interaction between SDSn and SDSn ASP in system A as shown in Figure 7. The interaction between SDSn molecules is mainly dominated by van der Waals. In the first 8 ns, the van der Waals attraction decreases rapidly from -2400 kJ/mol to -800 kJ/mol, which corresponds to the micelle dispersion process. Thereafter, the interaction between SDSn molecules is stable at about −800 kJ/mol, indicating that SDSn interactions are not the main driving force for adsorption and migration of SDSn on the surface of the oil droplet (Figure 7a). It can be seen that before 28 ns, van der Waals interaction between SDSn and asphaltene attracts increasingly, and it is divided into two stages according to the growth rate: During 0–8 ns, the interaction rapidly changes from 0 to −800 kJ/mol, the growth rate of 8–28 ns slows down, from −800 to −889 kJ/mol, which means that in the process of micelles approaching oil droplet, the van der Waals interaction between the SDSn molecules and asphaltenes increases rapidly, then the process of decentralization and migration also increases (Figure 7b). The Coulomb interaction has a small proportion and a small increase, which shows that the van der Waals attractive interaction between SDSn and asphaltenes is the main driving force during the whole process of aggregation (Figure 7a,b). That is to say, SDSn molecules are more likely to be combined with asphaltenes containing carboxylate than to aggregate with themselves and the attraction between SDSn molecules and asphaltenes containing carboxylate in system A can further emulsify by penetrating deep into oil droplet.

### 2.3. Emulsification Mechanism

**Figure 8:**
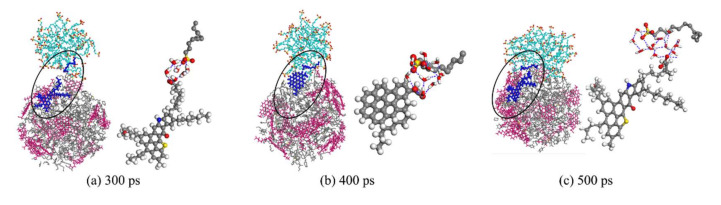
Partial amplification of hydrogen bond between SDSn and asphaltene at (**a**) 300 ps, (**b**) 400 ps, (**c**) 500 ps.

In system A, the enlarged picture of SDSn molecules interacting with acidified asphaltene molecules to spread on the surface of the oil droplet is shown in Figure 8. During the adsorption of surfactant by oil droplet, both the SDSn and asphaltene polarity heads are oriented towards the water molecules. Due to the saturation and directionality of the hydrogen bond, it can be seen that the two polar heads always take the solvent layer of water molecules as the medium and do not directly form the hydrogen bond. This means that in the process of adsorption, there is no superposition and aggregation between surfactant and asphaltenes. On the contrary, the two active substances are dispersed on the surface of oil droplets as much as possible, even when SDSn molecules migrate and evenly distribute the oil surface. The polar head of SDSn and asphaltenes hardly contacted directly, but through the water molecule as a water bridge (Figure 8) to COO^−^ polar head water molecule polarity SO^3-^ form the hydrogen bonding interaction. Under the pulling of asphaltenes, the water bridge is used as a bond to continuously slide the SDSn to the surface of the oil droplet.

The potential of mean force (PMF) between the group atom and the water molecule can be used to characterize the strength of its interaction with the water molecule and the stability of the hydration layer, and explain the energy barrier to be overcome when the group atom interacts with other molecules or ions [34]. The formula is calculated by the radial distribution function g(r) between it and water molecules, through the formula (2)

(2) $$E\left(r\right)=-k_{B}T\ln g\left(r\right)$$

**Figure 9:**
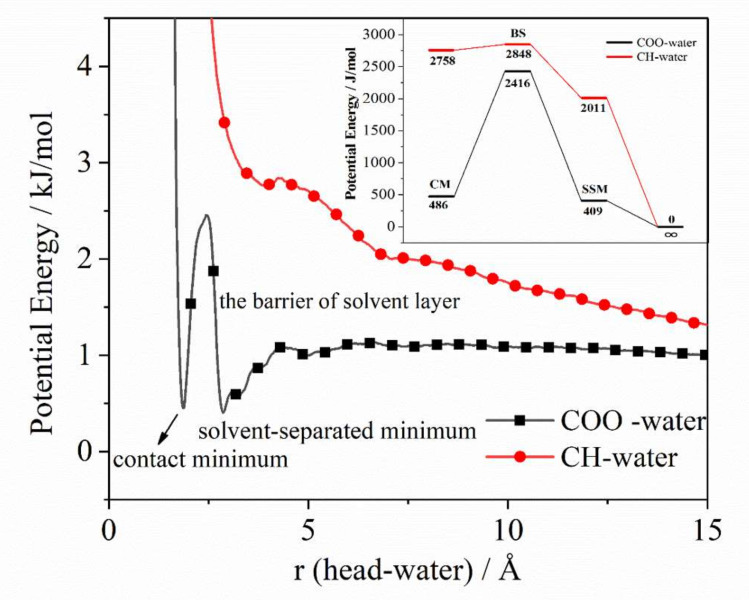
The PMF between the groups and the water molecule.

PMF is obtained, where *k_B_* is the Boltzmann constant and *T* is the temperature of the simulated system. The acidified molecular groups in system A and the unacidified molecular groups in system B were selected to calculate the PMF between the groups and the water molecule as shown in Figure 9, indicating the solvent layer energy barrier to be overcome in the process of external molecules or ions approaching the group. We take the COO curve as an example to illustrate a few points: 1) The minimum point of the PMF potential energy curve (CM, contact minimum) is about 0.19 nm, indicating the direct contact distance between the polar head and water molecules; 2) the second minimum point of the potential energy curve is about 0.28 nm, which is the solvent-separated minimum point (SSM), indicating the position where the second solvent layer is in contact with water molecules. The energy values corresponding to CM and SSM determine the stability of the combination of water molecules and polar heads in the first hydration layer and the second hydration layer; 3) there is a relatively high barrier of a solvent layer (BS) between CM and SSM. It means that the energy barrier other molecules have to overcome when they enter the first hydration layer from the second hydration layer of the polar head and unite with the polar hydrophilic group. It also explains the stability of the water substructure around the polar head.

The binding energy between groups and water molecules is determined by SSM and BS, namely ΔE^+^ = EBS − ESSM; and its dissociation energy depends on CM and BS, namely ΔE^−^ = EBS − ECM. Table 1 shows the binding energy and dissociation energy between each group and water. Combined with Figure 9 and Table 1, the following conclusions can be drawn: (1) A relatively stable energy hydration layer is formed between COO and water molecules, corresponding to the energy in the first hydration layer 486 J/mol, which is far less than that of unacidified groups 2758 J/mol, (2) the binding energy of COO with water ΔE^+^ (2.007 kJ/mol) is greater than that of unacidified groups with water ΔE^+^ (0.837 kJ/mol), indicating that the energy barrier to be overcome by the combination of COO and water molecules is higher, which also shows that the combination of COO and water molecules is more difficult for unacidified groups. We also notice that the dissociation energy of COO and water molecules ΔE^−^ (1.930 kJ/mol) is much larger than that of unacidified groups and water molecules ΔE^−^ (0.09 kJ/mol), which means that although it is difficult for SDSn molecules to cross the solvent layer energy barrier and combine water molecules, once the bonding pair and the hydration layer is formed, the formed hydration layer is difficult to disintegrate extremely stable. It can be obtained from the above analysis that the combination of water molecules and COO is difficult, but once the water molecules form a solvent layer, it is also stable. In contrast, the hydration layer formed by the unacidified groups are unstable, and the desorption and adsorption are very rapid.

**Figure 10:**
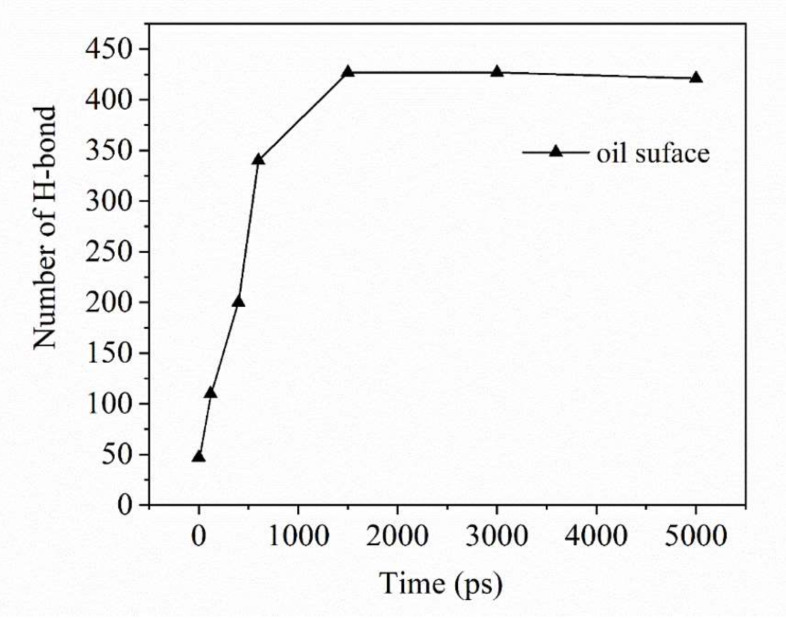
The number of H-bond as a function of time between the surface of oil droplet and water in system A.

The number of hydrogen bonds contains a lot of rich information. The existence of hydrogen bonds in molecular simulation can be judged by the angle formed by the hydrogen bond donor-H atom-hydrogen bond acceptor and the distance between the donor and acceptor atoms. Hydration layer can be formed around SDSn, and carboxylic acids on ASP on the surface of oil droplets in system A can also form hydration layer, thus, to understand the emulsification of the oil droplet surface, the molecular dynamics simulation calculated the number of hydrogen bonds between the oil droplet surface and water in the 0–5 ns emulsification process of system A (Figure 10). Since both the polar heads COO^−^ and SO_3_^−^ are easy to form hydrogen bonds with water molecules, the number of hydrogen bonds forms between the surface of oil droplets and water molecules increases rapidly from 47 at 0 ns to 427 at 1,500 ps and then keep 427 floating up and down. According to the PMF and the number of hydrogen bonds, we know that the hydration layer on the surface of the oil droplet in System A is stable and the polarization ability is strong, while the hydration layer in System B is unstable and its polarization is weak. SDSn molecules are easy to form hydrogen bond with the oil droplet of the more stable hydration layer in System A, and then aggregate with oil droplet, while that is more difficult in system B. It also proves from the side that the oil droplets attached to the surfactant can form a large number of hydrogen bonds with the aqueous solution, thereby promoting the stability of the oil-in-water emulsion and achieving the emulsification effect.

## 3. Materials and Methods

### 3.1. Simulation and Force Field

Molecular dynamics simulations were performed using the GROMACS (2019.4) [35,36] software package, and the GROMOS 54A8 force field was selected [37]. The simulated potential energy functions include bond length, bond angle, dihedral angle, and other bonding potential and non-bonding interaction potential, in which non-bonding interactions includes Lennard Jones potential and Coulomb interaction potential. The calculation formula of the simulated potential energy function is shown in Equation (3)

(3) $$U_{ij}\left(r\right)=4\varepsilon_{ij}\left[{\left(\frac{\sigma_{ij}}{r_{ij}}\right)}^{12}-{\left(\frac{\sigma_{ij}}{r_{ij}}\right)}^{6}\right]+\frac{q_{i}q_{j}}{r_{ij}}$$

In Equation (3), $r_{ij}$ is the distance between atoms i and j, $q_{i}$ is the charge assigned to the ith atom, $\sigma_{ij}$ is related to the equilibrium distance between i and j, and $\varepsilon_{ij}$ is the intensity of action. The structure parameters of the heavy oil molecules and the surfactant SDSn were generated by the Automated Topology Builder (ATB) and Repository [38,39] databases, and water molecules were selected the simple point charge (SPC) model [40]. The main force field parameters and charges in the simulation are shown in Table S1–S19.

### 3.2. Molecular Model

#### 3.2.1. Heavy Oil Model

**Figure 11:**
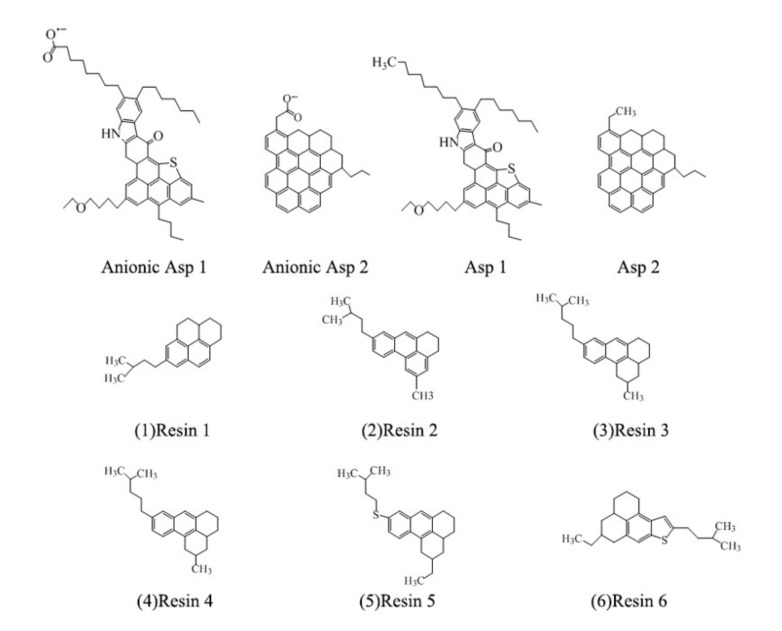
Asphaltenes and Resins used in the simulation. System A contains anionic Asp 1 and 2, and system B contains Asp 1 and 2, respectively.

The heavy oil droplet is composed of 2 types of asphaltenes [41], 6 types of resins [42] (Figure 11) and 8 types of alkanes. The types of alkanes are based on the crude oil model of Miranda [28,43]. To be close to the “real” heavy oil, the resins and asphaltenes accounted for 38% of the total mass of heavy oil droplets. According to the oxygen content of asphaltene molecules and whether they contained carboxylate groups or not, the simulated systems are divided into systems A and B, as shown in Table 1. The former was the system containing carboxylated groups.

**Figure 12:**
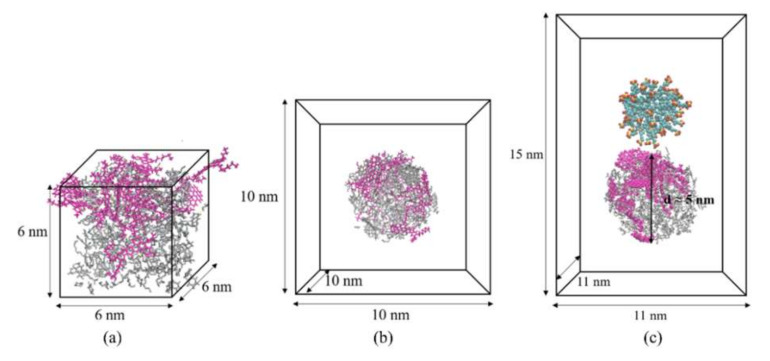
Simulation of the initial structure and dimensions. (**a**) Crude oil phase. (**b**) Heavy oil droplet. (**c**) Emulsified oil droplet. To be shown clearly, water molecules were removed in Figure (b) and (c).

At the initial simulation, asphaltenes, resins and alkanes were randomly placed in 10 × 10 × 10 nm^3^ cubic box (see Table 2), and in order to maintain its electrical neutrality, some Na^+^ ions were added to system A. The constant-pressure, constant-temperature (NPT) ensemble was operated at a temperature of 300 K and a pressure of 0.1 MPa to achieve a suitable density. At last, one 6 × 6 × 6 nm^3^ cubic box containing crude oil phase was obtained (see Figure 12a).

#### 3.2.2. Heavy Oil Droplets and Micelle Model

SDSn micelles with a concentration greater than critical micelle concentration (CMC) were added to the oil-in-water emulsion, assuming that a micelle and small oil droplets were captured from the macro solution. After one reasonable density of the heavy oil was obtained from NPT ensemble, the heavy oil system was put into the center of another 10 × 10 × 10 nm^3^ cubic box filled with simple point charge (SPC) water molecules. Then, the 30 ns canonical ensemble (NVT) ensemble was run to obtain one heavy oil droplet model surrounded by water molecules (see Figure 12b). Meanwhile, one spherical micelle of SDSn surfactant containing 50 molecules was obtained from another NPT ensemble simulation in 6 × 6 × 6 nm^3^ box filled with SPC water molecules according to the similar simulation above.

#### 3.2.3. Emulsified Oil Droplet Model

To study the formation and mechanism of emulsified oil droplets, SDSn micelles were used to mix with heavy oil droplets in 11 × 11 × 15 nm^3^ box (see Figure 12c). Based on the difference of asphaltenes, system A and system B were established respectively. And the initial simulated model was shown in Figure 12. Whether the oil droplets can be emulsified is related to the position of the micelles. Considering the configuration of heavy oil during real emulsification, the distance between micelles and heavy oil droplets is within the energy barrier, which is less than 2 nm, based on the Derjaguin Landau Verwey Overbeek (DLVO) theory [44]. The boxes were filled with SPC water molecules to run NPT ensemble for at least 50 ns. The details of the simulation system are listed in Table 3.

### 3.3. Molecular Dynamics Simulation

After the simulated model was constructed, the steepest descent method was set to minimize the energy, and the energy less than 1000 kJ∙mol^−1^nm^−1^ was set to reach the convergence standard. The simulated temperature and pressure of the NPT system were set to 300 K and 0.1 MPa, respectively. The time step was set to 2 fs, and the periodic boundary condition [45] was used. In the simulation, the bond length was constrained by the Linear Constraint Solver (LINCS) algorithm [35]. Velocity rescaling thermostat [46] was chosen with a time constant of 0.1 ps as the temperature coupling method. The Berendsen pressure coupling [47] and the adjustment time constant as 1.0 ps were selected, and the isothermal compression was adjusted to 4.5 × 10^−5^·bar^−1^. In the simulation, the cutoff distance of Lennard Jones potential interactions was set to 1.4 nm. For coulomb interaction, the summation method of particle-mesh Ewald (PME) was selected [48,49]. The Verlet list was updated every 10 steps, and the initial atomic velocity of the system was determined by the Maxwell Boltzmann distribution [42]. The entire trajectory is integrated by the leapfrog Verlet algorithm [50]. The dynamics properties were analyzed using the built-in analytical tools in GROMACS, and the trajectory was observed with VMD1.8.9.

## 4. Conclusions

**Figure 13:**
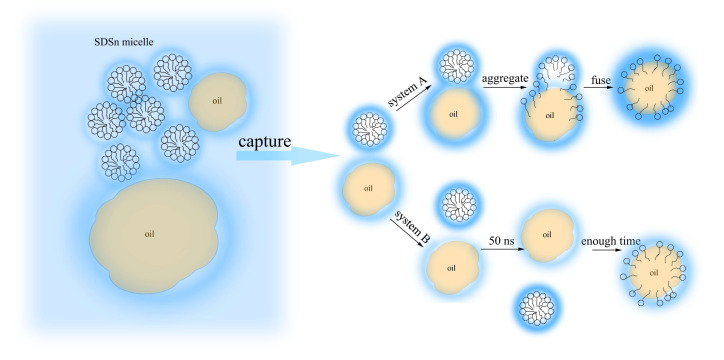
Schematic diagram of SDSn emulsified oil droplets in system A and B.

The emulsification process of the oil-water emulsion was studied by molecular dynamics simulation when SDSn micelles were mixed with two types of oil droplets. Since the asphaltenes containing carboxylate groups were added to the oil droplet in system A, it makes the hydration layer of the oil droplet more stable and highly hydrophilic. Under the interaction of van der Waals, by hydrogen bonds with water molecules to form a hydration layer, the hydration layer becomes a slip link of the asphaltene molecules and SDSn which form a water bridge structure to attract their aggregation and fusion. However, the hydration layer formed by the oil droplets without hydrophilic groups in system B is unstable, and SDSn molecules are not easy to merge into the oil droplet (Figure 13). Therefore, changing the hydrophilic/lipophilic of the oil droplets and increasing the hydrophilicity of the oil surface will promote emulsification.

## Figures and Tables

**Table 1:** The binding energy and dissociation energy between the groups and the water molecule.

Groups	△E^+^ (kJ/mol)	△E^—^ (kJ/mol)
COO	2.007	1.930
CH	0.837	0.090

**Table 2:** Details of the heavy oil simulation system.

Oil Droplet Type		A	B
Asphalt	Anionic Asp 1	32	0 0
	Anionic Asp 2	32	
	Asp 1	0 0	32
	Asp 2		32
Cation	Na^+^	64	0
Hydrocarbon	Hexane	256	256
	Heptane	236	236
	Octane	276	276
	Nonane	320	320
	Cyclohexane	172	172
	Toluene	276	276
	Benzene	108	108
Resin	Resin 1	40	40
	Resin 2 Resin 3	40 40	40 40
	Resin 4	40	40
	Resin 5	40	40
	Resin 6	40	40

**Table 3:** Details of crude oil emulsion simulation system.

System	SDSn Number	Na^+^ Number	Water Number	Box Size (nm^3^)
A	50	67	56,410	11 × 11 × 15
B	50	50	57,609	11 × 11 × 15

## Supplementary Materials

The following are available online: Figure S1: RMSD of SDSn in water, Figure S2: Snapshots of the system B at different time in NPT simulation, Figure S3: Velocity of SDSn relative to the oil drop in water, Figure S4: Velocity of SDSn relative to the oil drop in water, Table S1–Table S19: The field parameters of molecules used in the simulation.
