# Supplementary material for: Emulsification of Surfactant on Oil Droplets by Molecular Dynamics Simulation
Source: Molecules. 2020 Jun 30;25(13):3008. doi: 10.3390/molecules25133008 (PMC7412001; doi:10.3390/molecules25133008)
Supplement: Supplementary file 1 [file molecules-25-03008-s001.pdf]

Supplementary Materials

Emulsification of Surfactant on Oil Droplets by Molecular Dynamics Simulation

Yaoshuang Cheng and Shiling Yuan *

Key Lab of Colloid and Interface Chemistry, Shandong University, Jinan 250199, China; yaoshuangcheng@mail.sdu.edu.cn

* Correspondence:Correspondence: shilingyuan@sdu.edu.cn; Tel.: +86-531-88365896

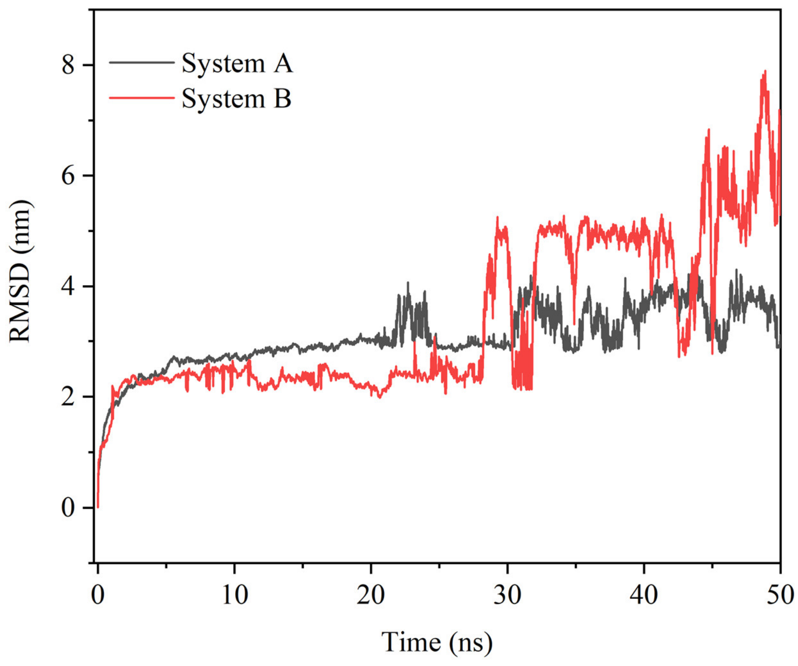

Figure S1. RMSD of SDSn in water.

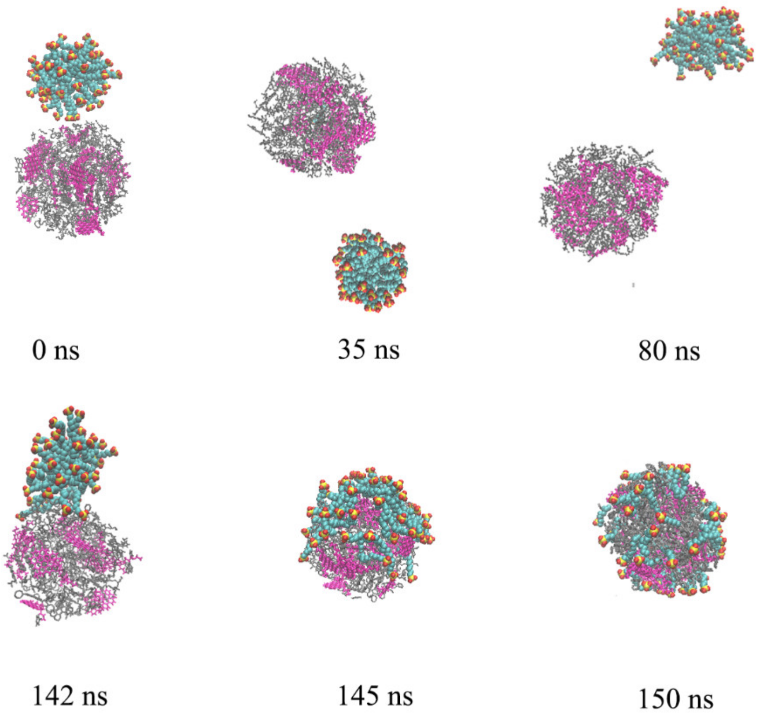

Figure S2. Snapshots of the system B at different time in NPT simulation. SDSn are displayed in blue, yellow and red spheres, ASP are displayed in rods and marked with rose red, and other heavy oil molecules are marked in gray. To be shown clearly, sodium ions and water molecules are removed.

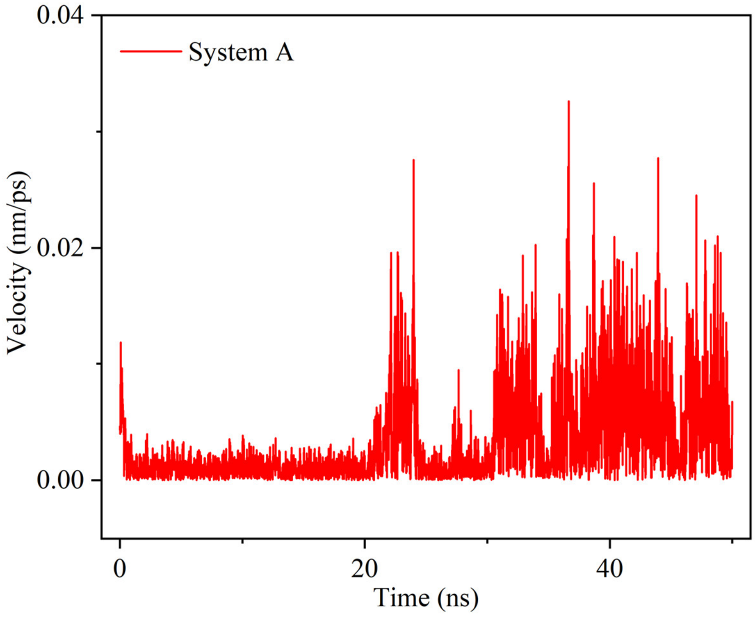

Figure S3. Velocity of SDSn relative to the oil drop in water.

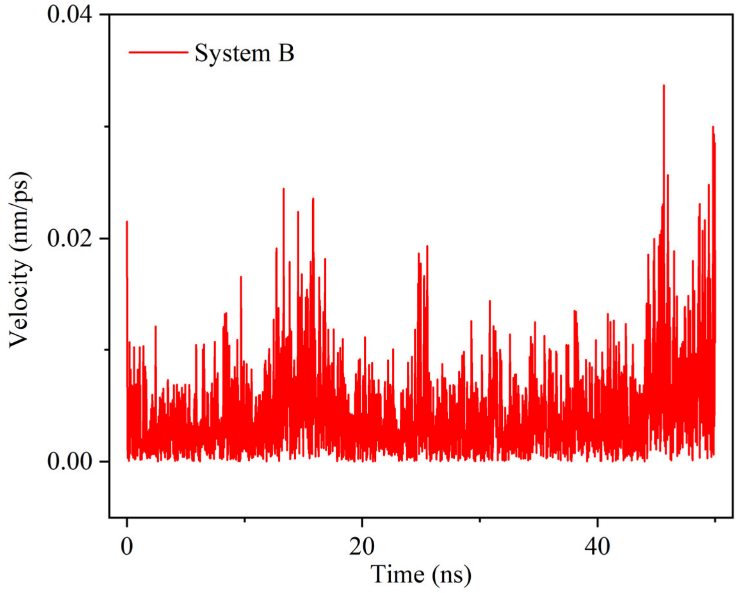

Figure S4. Velocity of SDSn relative to the oil drop in water.

Table S1. The field parameters of Anionic Asp 1 used in simulation.

Atom Type	C6	C12	Charge
OM	0.0022619536	7.4149321e-07	-0.588
C	0.0023406244	4.937284e-06	0.283
OM	0.0022619536	7.4149321e-07	-0.588
C	0.0023406244	4.937284e-06	-0.223
HC	8.464e-05	1.5129e-08	0.058
HC	8.464e-05	1.5129e-08	0.058
C	0.0023406244	4.937284e-06	-0.135
HC	8.464e-05	1.5129e-08	0.083
HC	8.464e-05	1.5129e-08	0.083
C	0.0023406244	4.937284e-06	-0.157
HC	8.464e-05	1.5129e-08	0.065
HC	8.464e-05	1.5129e-08	0.065
C	0.0023406244	4.937284e-06	-0.158
HC	8.464e-05	1.5129e-08	0.077
HC	8.464e-05	1.5129e-08	0.077
C	0.0023406244	4.937284e-06	-0.159
HC	8.464e-05	1.5129e-08	0.074
HC	8.464e-05	1.5129e-08	0.074
C	0.0023406244	4.937284e-06	-0.153
HC	8.464e-05	1.5129e-08	0.082
HC	8.464e-05	1.5129e-08	0.082
C	0.0023406244	4.937284e-06	-0.13
HC	8.464e-05	1.5129e-08	0.088
HC	8.464e-05	1.5129e-08	0.088
C	0.0023406244	4.937284e-06	-0.035
C	0.0023406244	4.937284e-06	-0.148
HC	8.464e-05	1.5129e-08	0.137
C	0.0023406244	4.937284e-06	-0.067
C	0.0023406244	4.937284e-06	-0.122
HC	8.464e-05	1.5129e-08	0.09

HC	8.464e-05	1.5129e-08	0.09
C	0.0023406244	4.937284e-06	-0.153
HC	8.464e-05	1.5129e-08	0.081
HC	8.464e-05	1.5129e-08	0.081
C	0.0023406244	4.937284e-06	-0.08
HC	8.464e-05	1.5129e-08	0.119
C	0.0023406244	4.937284e-06	-0.039
C	0.0023406244	4.937284e-06	-0.194
C	0.0023406244	4.937284e-06	0.051
NT	0.0024364096	5.0625e-06	-0.2
HS14	0.0	0.0	0.341
C	0.0023406244	4.937284e-06	0.002
C	0.0023406244	4.937284e-06	0.331
O	0.0022619536	1e-06	-0.331
C	0.0023406244	4.937284e-06	-0.125
C	0.0023406244	4.937284e-06	0.021
C	0.0023406244	4.937284e-06	-0.131
HC	8.464e-05	1.5129e-08	0.235
C	0.0023406244	4.937284e-06	-0.022
C	0.0023406244	4.937284e-06	0.018
C	0.0023406244	4.937284e-06	-0.098
HC	8.464e-05	1.5129e-08	0.102
C	0.0023406244	4.937284e-06	-0.033
C	0.0023406244	4.937284e-06	-0.018
C	0.0023406244	4.937284e-06	-0.133
HC	8.464e-05	1.5129e-08	0.092
HC	8.464e-05	1.5129e-08	0.092
C	0.0023406244	4.937284e-06	-0.112
HC	8.464e-05	1.5129e-08	0.127
C	0.0023406244	4.937284e-06	-0.071
C	0.0023406244	4.937284e-06	-0.124
HC	8.464e-05	1.5129e-08	0.09
HC	8.464e-05	1.5129e-08	0.09
C	0.0023406244	4.937284e-06	-0.195
HC	8.464e-05	1.5129e-08	0.086
HC	8.464e-05	1.5129e-08	0.086
C	0.0023406244	4.937284e-06	-0.163
HC	8.464e-05	1.5129e-08	0.093
HC	8.464e-05	1.5129e-08	0.093
C	0.0023406244	4.937284e-06	0.042
HC	8.464e-05	1.5129e-08	0.066
HC	8.464e-05	1.5129e-08	0.066
OE	0.0022619536	1.21e-06	-0.284
C	0.0023406244	4.937284e-06	-0.02
HC	8.464e-05	1.5129e-08	0.065
HC	8.464e-05	1.5129e-08	0.065
C	0.0023406244	4.937284e-06	-0.252
HC	8.464e-05	1.5129e-08	0.084
HC	8.464e-05	1.5129e-08	0.084
HC	8.464e-05	1.5129e-08	0.084
C	0.0023406244	4.937284e-06	-0.015
C	0.0023406244	4.937284e-06	-0.045
C	0.0023406244	4.937284e-06	-0.11
HC	8.464e-05	1.5129e-08	0.17
C	0.0023406244	4.937284e-06	-0.281
S	0.0099840064	1.3075456e-05	0.525
C	0.0023406244	4.937284e-06	-0.165

C	0.0023406244	4.937284e-06	-0.079
C	0.0023406244	4.937284e-06	-0.068
HC	8.464e-05	1.5129e-08	0.064
C	0.0023406244	4.937284e-06	-0.069
C	0.0023406244	4.937284e-06	-0.182
HC	8.464e-05	1.5129e-08	0.085
HC	8.464e-05	1.5129e-08	0.085
HC	8.464e-05	1.5129e-08	0.085
C	0.0023406244	4.937284e-06	-0.184
HC	8.464e-05	1.5129e-08	0.089
HC	8.464e-05	1.5129e-08	0.089
C	0.0023406244	4.937284e-06	-0.159
HC	8.464e-05	1.5129e-08	0.077
HC	8.464e-05	1.5129e-08	0.077
C	0.0023406244	4.937284e-06	-0.211
HC	8.464e-05	1.5129e-08	0.074
HC	8.464e-05	1.5129e-08	0.074
HC	8.464e-05	1.5129e-08	0.074
C	0.0023406244	4.937284e-06	-0.161
HC	8.464e-05	1.5129e-08	0.083
HC	8.464e-05	1.5129e-08	0.083
C	0.0023406244	4.937284e-06	-0.159
HC	8.464e-05	1.5129e-08	0.077
HC	8.464e-05	1.5129e-08	0.077
C	0.0023406244	4.937284e-06	-0.156
HC	8.464e-05	1.5129e-08	0.08
HC	8.464e-05	1.5129e-08	0.08
C	0.0023406244	4.937284e-06	-0.159
HC	8.464e-05	1.5129e-08	0.077
HC	8.464e-05	1.5129e-08	0.077
C	0.0023406244	4.937284e-06	-0.209
HC	8.464e-05	1.5129e-08	0.07
HC	8.464e-05	1.5129e-08	0.07
HC	8.464e-05	1.5129e-08	0.07

Table S2. The field parameters of Anionic Asp 2 used in simulation.

Atom Type	C6	C12	Charge
O	0.0022619536	1e-06	0.035
C	0.0023406244	4.937284e-06	-0.106
HC	8.464e-05	1.5129e-08	0.035
HC	8.464e-05	1.5129e-08	0.035
C	0.0023406244	4.937284e-06	-0.108
HC	8.464e-05	1.5129e-08	0.048
HC	8.464e-05	1.5129e-08	0.061
C	0.0023406244	4.937284e-06	-0.11
HC	8.464e-05	1.5129e-08	0.059
HC	8.464e-05	1.5129e-08	0.056
C	0.0023406244	4.937284e-06	-0.034
HC	8.464e-05	1.5129e-08	0.07
C	0.0023406244	4.937284e-06	-0.042
C	0.0023406244	4.937284e-06	-0.127
HC	8.464e-05	1.5129e-08	0.128
C	0.0023406244	4.937284e-06	-0.018
C	0.0023406244	4.937284e-06	-0.011
C	0.0023406244	4.937284e-06	-0.057

C	0.0023406244	4.937284e-06	-0.03
C	0.0023406244	4.937284e-06	-0.007
C	0.0023406244	4.937284e-06	-0.034
C	0.0023406244	4.937284e-06	-0.095
HC	8.464e-05	1.5129e-08	0.151
C	0.0023406244	4.937284e-06	-0.099
HC	8.464e-05	1.5129e-08	0.077
C	0.0023406244	4.937284e-06	-0.043
C	0.0023406244	4.937284e-06	-0.103
HC	8.464e-05	1.5129e-08	0.097
C	0.0023406244	4.937284e-06	-0.087
HC	8.464e-05	1.5129e-08	0.087
C	0.0023406244	4.937284e-06	0
C	0.0023406244	4.937284e-06	-0.003
C	0.0023406244	4.937284e-06	-0.015
C	0.0023406244	4.937284e-06	0.018
C	0.0023406244	4.937284e-06	-0.047
C	0.0023406244	4.937284e-06	-0.102
HC	8.464e-05	1.5129e-08	0.098
C	0.0023406244	4.937284e-06	-0.096
HC	8.464e-05	1.5129e-08	0.147
C	0.0023406244	4.937284e-06	-0.047
C	0.0023406244	4.937284e-06	-0.13
HC	8.464e-05	1.5129e-08	0.099
C	0.0023406244	4.937284e-06	-0.091
HC	8.464e-05	1.5129e-08	0.169
C	0.0023406244	4.937284e-06	-0.028
C	0.0023406244	4.937284e-06	-0.022
C	0.0023406244	4.937284e-06	-0.191
HC	8.464e-05	1.5129e-08	0.057
HC	8.464e-05	1.5129e-08	0.148
C	0.0023406244	4.937284e-06	-0.016
HC	8.464e-05	1.5129e-08	0.083
C	0.0023406244	4.937284e-06	-0.031
C	0.0023406244	4.937284e-06	-0.071
C	0.0023406244	4.937284e-06	-0.026
HC	8.464e-05	1.5129e-08	0.083
C	0.0023406244	4.937284e-06	-0.107
HC	8.464e-05	1.5129e-08	0.064
HC	8.464e-05	1.5129e-08	0.055
C	0.0023406244	4.937284e-06	-0.097
HC	8.464e-05	1.5129e-08	0.042
HC	8.464e-05	1.5129e-08	0.057
C	0.0023406244	4.937284e-06	-0.1
HC	8.464e-05	1.5129e-08	0.05
HC	8.464e-05	1.5129e-08	0.05
C	0.0023406244	4.937284e-06	-0.005
C	0.0023406244	4.937284e-06	-0.157
HC	8.464e-05	1.5129e-08	0.052
HC	8.464e-05	1.5129e-08	0.051
C	0.0023406244	4.937284e-06	0.441
OM	0.0022619536	7.4149321e-07	-0.605
OM	0.0022619536	7.4149321e-07	-0.605

Table S3. The field parameters of Asp 1 used in simulation.

Atom Type	C6	C12	Charge
HC	8.464e-05	1.5129e-08	0.075
C	0.0023406244	4.937284e-06	-0.211
HC	8.464e-05	1.5129e-08	0.075
HC	8.464e-05	1.5129e-08	0.075
C	0.0023406244	4.937284e-06	-0.159
HC	8.464e-05	1.5129e-08	0.078
HC	8.464e-05	1.5129e-08	0.078
C	0.0023406244	4.937284e-06	-0.189
HC	8.464e-05	1.5129e-08	0.089
HC	8.464e-05	1.5129e-08	0.089
C	0.0023406244	4.937284e-06	-0.136
HC	8.464e-05	1.5129e-08	0.094
HC	8.464e-05	1.5129e-08	0.094
C	0.0023406244	4.937284e-06	-0.013
C	0.0023406244	4.937284e-06	-0.039
C	0.0023406244	4.937284e-06	-0.103
HC	8.464e-05	1.5129e-08	0.122
C	0.0023406244	4.937284e-06	-0.076
C	0.0023406244	4.937284e-06	-0.125
HC	8.464e-05	1.5129e-08	0.091
HC	8.464e-05	1.5129e-08	0.091
C	0.0023406244	4.937284e-06	-0.089
HC	8.464e-05	1.5129e-08	0.094
C	0.0023406244	4.937284e-06	-0.029
C	0.0023406244	4.937284e-06	0.024
C	0.0023406244	4.937284e-06	-0.089
C	0.0023406244	4.937284e-06	-0.155
S	0.0099840064	1.3075456e-05	0.534
C	0.0023406244	4.937284e-06	-0.29
C	0.0023406244	4.937284e-06	-0.057
HC	8.464e-05	1.5129e-08	0.049
C	0.0023406244	4.937284e-06	-0.07
C	0.0023406244	4.937284e-06	-0.183
HC	8.464e-05	1.5129e-08	0.087
HC	8.464e-05	1.5129e-08	0.087
HC	8.464e-05	1.5129e-08	0.087
C	0.0023406244	4.937284e-06	-0.104
HC	8.464e-05	1.5129e-08	0.163
C	0.0023406244	4.937284e-06	-0.02
C	0.0023406244	4.937284e-06	-0.039
C	0.0023406244	4.937284e-06	0.039
C	0.0023406244	4.937284e-06	-0.14
C	0.0023406244	4.937284e-06	-0.15
HC	8.464e-05	1.5129e-08	0.188
C	0.0023406244	4.937284e-06	0.063
C	0.0023406244	4.937284e-06	0.485
O	0.0022619536	1e-06	-0.335
C	0.0023406244	4.937284e-06	-0.198
C	0.0023406244	4.937284e-06	-0.035
C	0.0023406244	4.937284e-06	-0.075
HC	8.464e-05	1.5129e-08	0.158

C	0.0023406244	4.937284e-06	0
NT	0.0024364096	5.0625e-06	-0.205
S14	0.0	0.0	0.22
C	0.0023406244	4.937284e-06	-0.149
HC	8.464e-05	1.5129e-08	0.134
C	0.0023406244	4.937284e-06	-0.064
C	0.0023406244	4.937284e-06	-0.125
HC	8.464e-05	1.5129e-08	0.092
HC	8.464e-05	1.5129e-08	0.092
C	0.0023406244	4.937284e-06	-0.157
HC	8.464e-05	1.5129e-08	0.081
HC	8.464e-05	1.5129e-08	0.081
C	0.0023406244	4.937284e-06	-0.057
C	0.0023406244	4.937284e-06	-0.121
HC	8.464e-05	1.5129e-08	0.086
HC	8.464e-05	1.5129e-08	0.086
C	0.0023406244	4.937284e-06	-0.152
HC	8.464e-05	1.5129e-08	0.079
HC	8.464e-05	1.5129e-08	0.079
C	0.0023406244	4.937284e-06	-0.162
HC	8.464e-05	1.5129e-08	0.086
HC	8.464e-05	1.5129e-08	0.086
C	0.0023406244	4.937284e-06	-0.162
HC	8.464e-05	1.5129e-08	0.076
HC	8.464e-05	1.5129e-08	0.076
C	0.0023406244	4.937284e-06	-0.159
HC	8.464e-05	1.5129e-08	0.081
HC	8.464e-05	1.5129e-08	0.081
C	0.0023406244	4.937284e-06	-0.158
HC	8.464e-05	1.5129e-08	0.076
HC	8.464e-05	1.5129e-08	0.076
C	0.0023406244	4.937284e-06	-0.21
HC	8.464e-05	1.5129e-08	0.071
HC	8.464e-05	1.5129e-08	0.071
HC	8.464e-05	1.5129e-08	0.071
C	0.0023406244	4.937284e-06	-0.159
HC	8.464e-05	1.5129e-08	0.08
HC	8.464e-05	1.5129e-08	0.08
C	0.0023406244	4.937284e-06	-0.159
HC	8.464e-05	1.5129e-08	0.079
HC	8.464e-05	1.5129e-08	0.079
C	0.0023406244	4.937284e-06	-0.158
HC	8.464e-05	1.5129e-08	0.079
HC	8.464e-05	1.5129e-08	0.079
C	0.0023406244	4.937284e-06	-0.159
HC	8.464e-05	1.5129e-08	0.078
HC	8.464e-05	1.5129e-08	0.078
C	0.0023406244	4.937284e-06	-0.159
HC	8.464e-05	1.5129e-08	0.078
HC	8.464e-05	1.5129e-08	0.078
C	0.0023406244	4.937284e-06	-0.21
HC	8.464e-05	1.5129e-08	0.072
HC	8.464e-05	1.5129e-08	0.072
HC	8.464e-05	1.5129e-08	0.072

C	0.0023406244	4.937284e-06	-0.194
HC	8.464e-05	1.5129e-08	0.085
HC	8.464e-05	1.5129e-08	0.085
C	0.0023406244	4.937284e-06	-0.164
HC	8.464e-05	1.5129e-08	0.094
HC	8.464e-05	1.5129e-08	0.094
C	0.0023406244	4.937284e-06	0.047
HC	8.464e-05	1.5129e-08	0.065
HC	8.464e-05	1.5129e-08	0.065
OE	0.0022619536	1.21e-06	-0.283
C	0.0023406244	4.937284e-06	-0.02
HC	8.464e-05	1.5129e-08	0.063
HC	8.464e-05	1.5129e-08	0.063
C	0.0023406244	4.937284e-06	-0.252
HC	8.464e-05	1.5129e-08	0.084
HC	8.464e-05	1.5129e-08	0.084
HC	8.464e-05	1.5129e-08	0.084

Table S4. The field parameters of Asp 2 used in simulation.

Atom Type	C6	C12	Charge
HC	8.464e-05	1.5129e-08	0.134
C	0.0023406244	4.937284e-06	-0.114
C	0.0023406244	4.937284e-06	-0.115
HC	8.464e-05	1.5129e-08	0.134
C	0.0023406244	4.937284e-06	-0.027
C	0.0023406244	4.937284e-06	-0.12
HC	8.464e-05	1.5129e-08	0.136
C	0.0023406244	4.937284e-06	-0.042
C	0.0023406244	4.937284e-06	-0.064
HC	8.464e-05	1.5129e-08	0.103
C	0.0023406244	4.937284e-06	-0.156
HC	8.464e-05	1.5129e-08	0.083
HC	8.464e-05	1.5129e-08	0.085
C	0.0023406244	4.937284e-06	-0.16
HC	8.464e-05	1.5129e-08	0.079
HC	8.464e-05	1.5129e-08	0.08
C	0.0023406244	4.937284e-06	-0.211
HC	8.464e-05	1.5129e-08	0.073
HC	8.464e-05	1.5129e-08	0.073
HC	8.464e-05	1.5129e-08	0.073
C	0.0023406244	4.937284e-06	-0.144
HC	8.464e-05	1.5129e-08	0.086
HC	8.464e-05	1.5129e-08	0.086
C	0.0023406244	4.937284e-06	-0.065
HC	8.464e-05	1.5129e-08	0.1
C	0.0023406244	4.937284e-06	-0.151
HC	8.464e-05	1.5129e-08	0.083
HC	8.464e-05	1.5129e-08	0.089
C	0.0023406244	4.937284e-06	-0.153
HC	8.464e-05	1.5129e-08	0.082
HC	8.464e-05	1.5129e-08	0.084

C	0.0023406244	4.937284e-06	-0.064
HC	8.464e-05	1.5129e-08	0.105
C	0.0023406244	4.937284e-06	-0.12
HC	8.464e-05	1.5129e-08	0.099
HC	8.464e-05	1.5129e-08	0.093
C	0.0023406244	4.937284e-06	-0.05
C	0.0023406244	4.937284e-06	-0.068
C	0.0023406244	4.937284e-06	-0.125
HC	8.464e-05	1.5129e-08	0.087
HC	8.464e-05	1.5129e-08	0.086
C	0.0023406244	4.937284e-06	-0.208
HC	8.464e-05	1.5129e-08	0.076
HC	8.464e-05	1.5129e-08	0.076
HC	8.464e-05	1.5129e-08	0.076
C	0.0023406244	4.937284e-06	-0.122
HC	8.464e-05	1.5129e-08	0.133
C	0.0023406244	4.937284e-06	-0.122
HC	8.464e-05	1.5129e-08	0.133
C	0.0023406244	4.937284e-06	-0.016
C	0.0023406244	4.937284e-06	-0.015
C	0.0023406244	4.937284e-06	-0.01
C	0.0023406244	4.937284e-06	-0.052
C	0.0023406244	4.937284e-06	-0.042
C	0.0023406244	4.937284e-06	-0.022
C	0.0023406244	4.937284e-06	-0.01
C	0.0023406244	4.937284e-06	-0.013
C	0.0023406244	4.937284e-06	-0.006
C	0.0023406244	4.937284e-06	-0.016
C	0.0023406244	4.937284e-06	-0.115
HC	8.464e-05	1.5129e-08	0.134
C	0.0023406244	4.937284e-06	-0.117
HC	8.464e-05	1.5129e-08	0.134
C	0.0023406244	4.937284e-06	-0.031
C	0.0023406244	4.937284e-06	-0.116
HC	8.464e-05	1.5129e-08	0.134
C	0.0023406244	4.937284e-06	-0.113
HC	8.464e-05	1.5129e-08	0.134
C	0.0023406244	4.937284e-06	-0.033
C	0.0023406244	4.937284e-06	-0.009
C	0.0023406244	4.937284e-06	-0.011
C	0.0023406244	4.937284e-06	-0.015

Table S5. The field parameters of Na^+ used in simulation.

Atom Type	C6	C12	Charge
NA+	7.2063121e-05	2.1025e-08	+1

Table S6. The field parameters of Hexane used in simulation.

Atom Type	C6	C12	Charge
CH3	0.0096138025	2.6646244e-05	-0.097
CH2	0.0074684164	3.3965584e-05	0.139
CH2	0.0074684164	3.3965584e-05	-0.042
CH2	0.0074684164	3.3965584e-05	-0.042

CH2	0.0074684164	3.3965584e-05	0.139
CH3	0.0096138025	2.6646244e-05	-0.097

Table S7. The field parameters of Heptane used in simulation.

Atom Type	C6	C12	Charge
CH3	0.0096138025	2.6646244e-05	-0.101
CH2	0.0074684164	3.3965584e-05	0.114
CH2	0.0074684164	3.3965584e-05	0.044
CH2	0.0074684164	3.3965584e-05	-0.114
CH2	0.0074684164	3.3965584e-05	0.044
CH2	0.0074684164	3.3965584e-05	0.114
CH3	0.0096138025	2.6646244e-05	-0.101

Table S8. The field parameters of Octane used in simulation.

Atom Type	C6	C12	Charge
CH3	0.0096138025	2.6646244e-05	-0.086
CH2	0.0074684164	3.3965584e-05	0.096
CH2	0.0074684164	3.3965584e-05	0.011
CH2	0.0074684164	3.3965584e-05	-0.021
CH2	0.0074684164	3.3965584e-05	-0.021
CH2	0.0074684164	3.3965584e-05	0.011
CH2	0.0074684164	3.3965584e-05	0.096
CH3	0.0096138025	2.6646244e-05	-0.086

Table S9. The field parameters of Nonane used in simulation.

Atom Type	C6	C12	Charge
CH3	0.0096138025	2.6646244e-05	-0.091
CH2	0.0074684164	3.3965584e-05	0.122
CH2	0.0074684164	3.3965584e-05	-0.017
CH2	0.0074684164	3.3965584e-05	-0.053
CH2	0.0074684164	3.3965584e-05	0.078
CH2	0.0074684164	3.3965584e-05	-0.053
CH2	0.0074684164	3.3965584e-05	-0.017
CH2	0.0074684164	3.3965584e-05	0.122
CH3	0.0096138025	2.6646244e-05	-0.091

Table S10. The field parameters of Cyclohexane used in simulation.

Atom Type	C6	C12	Charge
CH2	0.0074684164	3.3965584e-05	0
CH2	0.0074684164	3.3965584e-05	0
CH2	0.0074684164	3.3965584e-05	0
CH2	0.0074684164	3.3965584e-05	0
CH2	0.0074684164	3.3965584e-05	0
CH2	0.0074684164	3.3965584e-05	0

Table S11. The field parameters of Toluene used in simulation.

Atom Type	C6	C12	Charge
CH3	0.0096138025	2.6646244e-05	-0.076
C	0.0023406244	4.937284e-06	0.29
C	0.0023406244	4.937284e-06	-0.26
HC	8.464e-05	1.5129e-08	0.142
C	0.0023406244	4.937284e-06	-0.26
HC	8.464e-05	1.5129e-08	0.142
C	0.0023406244	4.937284e-06	-0.1
HC	8.464e-05	1.5129e-08	0.122
C	0.0023406244	4.937284e-06	-0.157
HC	8.464e-05	1.5129e-08	0.135
C	0.0023406244	4.937284e-06	-0.1
HC	8.464e-05	1.5129e-08	0.122

Table S12. The field parameters of Benzene used in simulation.

Atom Type	C6	C12	Charge
HC	8.464e-05	1.5129e-08	0.129
C	0.0023406244	4.937284e-06	-0.129
C	0.0023406244	4.937284e-06	-0.129
HC	8.464e-05	1.5129e-08	0.129
C	0.0023406244	4.937284e-06	-0.129
HC	8.464e-05	1.5129e-08	0.129
C	0.0023406244	4.937284e-06	-0.129
HC	8.464e-05	1.5129e-08	0.129
C	0.0023406244	4.937284e-06	-0.129
HC	8.464e-05	1.5129e-08	0.129
C	0.0023406244	4.937284e-06	-0.129
HC	8.464e-05	1.5129e-08	0.129

Table S13. The field parameters of Resin 1 used in simulation.

Atom Type	C6	C12	Charge
CH3	0.0096138025	2.6646244e-05	0.011
CH1	0.00606841	9.70225e-05	-0.019
CH2	0.0074684164	3.3965584e-05	0.019
CH2	0.0074684164	3.3965584e-05	0.054
C	0.0023406244	4.937284e-06	-0.064
C	0.0023406244	4.937284e-06	-0.122
HC	8.464e-05	1.5129e-08	0.132
C	0.0023406244	4.937284e-06	-0.128
HC	8.464e-05	1.5129e-08	0.133
C	0.0023406244	4.937284e-06	-0.066
C	0.0023406244	4.937284e-06	-0.023
CH2r	0.0073342096	2.8058209e-05	0.062
CH2r	0.0073342096	2.8058209e-05	0.020
CH1	0.00606841	9.70225e-05	0.022
C	0.0023406244	4.937284e-06	-0.051

CH2r	0.0073342096	2.8058209e-05	0.009
CH2r	0.0073342096	2.8058209e-05	0.009
CH2r	0.0073342096	2.8058209e-05	0.059
C	0.0023406244	4.937284e-06	-0.068
C	0.0023406244	4.937284e-06	-0.121
HC	8.464e-05	1.5129e-08	0.133
C	0.0023406244	4.937284e-06	-0.117
HC	8.464e-05	1.5129e-08	0.134
C	0.0023406244	4.937284e-06	-0.029
CH3	0.0096138025	2.6646244e-05	0.011

Table S14. The field parameters of Resin 2 used in simulation.

Atom Type	C6	C12	Charge
CH3	0.0096138025	2.6646244e-05	0.011
CH1	0.00606841	9.70225e-05	-0.011
CH3	0.0096138025	2.6646244e-05	0.011
CH2	0.0074684164	3.3965584e-05	0.018
CH2	0.0074684164	3.3965584e-05	0.051
C	0.0023406244	4.937284e-06	-0.069
C	0.0023406244	4.937284e-06	-0.115
HC	8.464e-05	1.5129e-08	0.13
C	0.0023406244	4.937284e-06	-0.026
C	0.0023406244	4.937284e-06	-0.132
HC	8.464e-05	1.5129e-08	0.13
C	0.0023406244	4.937284e-06	-0.055
CH2r	0.0073342096	2.8058209e-05	0.057
CH2r	0.0073342096	2.8058209e-05	0
CH2r	0.0073342096	2.8058209e-05	0.014
CH1	0.00606841	9.70225e-05	0.022
C	0.0023406244	4.937284e-06	-0.056
CH2r	0.0073342096	2.8058209e-05	0.02
CH1	0.00606841	9.70225e-05	-0.013
CH3	0.0096138025	2.6646244e-05	0.013
CH2r	0.0073342096	2.8058209e-05	0.064
C	0.0023406244	4.937284e-06	-0.048
C	0.0023406244	4.937284e-06	-0.034
C	0.0023406244	4.937284e-06	-0.118
HC	8.464e-05	1.5129e-08	0.136
C	0.0023406244	4.937284e-06	-0.128
HC	8.464e-05	1.5129e-08	0.128

Table S15. The field parameters of Resin 3 used in simulation.

Atom Type	C6	C12	Charge
CH3	0.0096138025	2.6646244e-05	0.011
CH1	0.00606841	9.70225e-05	-0.011
CH3	0.0096138025	2.6646244e-05	0.011
CH2	0.0074684164	3.3965584e-05	0.018
CH2	0.0074684164	3.3965584e-05	0.051
CH2	0.0074684164	3.3965584e-05	-0.069
C	0.0023406244	4.937284e-06	-0.115
C	0.0023406244	4.937284e-06	0.13
HC	8.464e-05	1.5129e-08	-0.026

C	0.0023406244	4.937284e-06	-0.132
HC	8.464e-05	1.5129e-08	0.13
C	0.0023406244	4.937284e-06	-0.055
C	0.0023406244	4.937284e-06	0.057
CH2r	0.0073342096	2.8058209e-05	0
CH1	0.00606841	9.70225e-05	0.014
CH3	0.0096138025	2.6646244e-05	0.022
CH2r	0.0073342096	2.8058209e-05	-0.056
CH1	0.00606841	9.70225e-05	0.02
C	0.0023406244	4.937284e-06	-0.013
CH2r	0.0073342096	2.8058209e-05	0.013
CH2r	0.0073342096	2.8058209e-05	0.064
CH2r	0.0073342096	2.8058209e-05	-0.048
C	0.0023406244	4.937284e-06	-0.034
C	0.0023406244	4.937284e-06	-0.118
HC	8.464e-05	1.5129e-08	0.136
C	0.0023406244	4.937284e-06	-0.128
C	0.0023406244	4.937284e-06	0.128
HC	8.464e-05	1.5129e-08	0.128

Table S16. The field parameters of Resin 4 used in simulation.

Atom Type	C6	C12	Charge
CH3	0.0096138025	2.6646244e-05	0
CH2	0.0074684164	3.3965584e-05	0.055
C	0.0023406244	4.937284e-06	-0.057
C	0.0023406244	4.937284e-06	-0.129
HC	8.464e-05	1.5129e-08	0.131
C	0.0023406244	4.937284e-06	-0.031
C	0.0023406244	4.937284e-06	-0.13
HC	8.464e-05	1.5129e-08	0.132
C	0.0023406244	4.937284e-06	-0.02
C	0.0023406244	4.937284e-06	-0.117
HC	8.464e-05	1.5129e-08	0.137
C	0.0023406244	4.937284e-06	-0.061
CH2r	0.0073342096	2.8058209e-05	0.061
CH1	0.00606841	9.70225e-05	-0.003
CH2r	0.0073342096	2.8058209e-05	0.008
CH1	0.00606841	9.70225e-05	-0.003
CH2r	0.0073342096	2.8058209e-05	0.008
CH2r	0.0073342096	2.8058209e-05	0.061
C	0.0023406244	4.937284e-06	-0.061
CH2r	0.0073342096	2.8058209e-05	0.003
CH2r	0.0073342096	2.8058209e-05	0.003
C	0.0023406244	4.937284e-06	-0.127
HC	8.464e-05	1.5129e-08	0.131
C	0.0023406244	4.937284e-06	-0.059
CH2	0.0074684164	3.3965584e-05	0.055
CH1	0.00606841	9.70225e-05	-0.013
CH3	0.0096138025	2.6646244e-05	0.013
CH3	0.0096138025	2.6646244e-05	0.013

Table S17. The field parameters of Resin 5 used in simulation.

Atom Type	C6	C12	Charge
CH3	0.0096138025	2.6646244e-05	-0.026
CH2	0.0074684164	3.3965584e-05	0.002
CH1	0.00606841	9.70225e-05	0.032
CH2	0.0074684164	3.3965584e-05	0.014
C	0.0023406244	4.937284e-06	-0.002
C	0.0023406244	4.937284e-06	0.082
CH1	0.00606841	9.70225e-05	0.032
CH2	0.0074684164	3.3965584e-05	-0.005
CH2	0.0074684164	3.3965584e-05	-0.011
C	0.0023406244	4.937284e-06	0.023
C	0.0023406244	4.937284e-06	-0.146
HC	8.464e-05	1.5129e-08	0.092
CH2	0.0074684164	3.3965584e-05	-0.008
C	0.0023406244	4.937284e-06	0.013
C	0.0023406244	4.937284e-06	0.03
C	0.0023406244	4.937284e-06	-0.149
HC	8.464e-05	1.5129e-08	0.088
C	0.0023406244	4.937284e-06	-0.081
HC	8.464e-05	1.5129e-08	0.137
C	0.0023406244	4.937284e-06	0.156
S	0.0099840064	1.3075456e-05	-0.486
C	0.0023406244	4.937284e-06	-0.095
HC	8.464e-05	1.5129e-08	0.086
CH2	0.0074684164	3.3965584e-05	0.118
CH2	0.0074684164	3.3965584e-05	0.131
CH1	0.00606841	9.70225e-05	0.005
CH3	0.0096138025	2.6646244e-05	-0.01
CH3	0.0096138025	2.6646244e-05	-0.003
CH2	0.0074684164	3.3965584e-05	-0.019

Table S18. The field parameters of Resin 6 used in simulation.

Atom Type	C6	C12	Charge
CH3	0.0096138025	2.6646244e-05	0
CH1	0.00606841	9.70225e-05	-0.021
CH2	0.0074684164	3.3965584e-05	0.017
CH2	0.0074684164	3.3965584e-05	0.004
C	0.0023406244	4.937284e-06	-0.317
S	0.0099840064	1.3075456e-05	0.606
C	0.0023406244	4.937284e-06	-0.296
C	0.0023406244	4.937284e-06	-0.146
HC	8.464e-05	1.5129e-08	0.153
C	0.0023406244	4.937284e-06	-0.098
HC	8.464e-05	1.5129e-08	0.094
C	0.0023406244	4.937284e-06	-0.057
CH2	0.0074684164	3.3965584e-05	0.061
C	0.0023406244	4.937284e-06	-0.063
CH1	0.00606841	9.70225e-05	0.031
CH2	0.0074684164	3.3965584e-05	0.013
CH2	0.0074684164	3.3965584e-05	0.019
CH2	0.0074684164	3.3965584e-05	0
CH2	0.0074684164	3.3965584e-05	0.089

C	0.0023406244	4.937284e-06	-0.023
C	0.0023406244	4.937284e-06	-0.066
CH1	0.00606841	9.70225e-05	-0.009
CH2	0.0074684164	3.3965584e-05	0.001
CH3	0.0096138025	2.6646244e-05	0.008
CH3	0.0096138025	2.6646244e-05	0

Table S19. The field parameters of SDSn used in simulation.

Atom Type	C6	C12	Charge
OM	0.0022619536	7.4149321e-07	-0.648
SDmso	0.010561673	2.149806e-05	0.844
CH2	0.0074684164	3.3965584e-05	0.083
CH2	0.0074684164	3.3965584e-05	-0.002
CH2	0.0074684164	3.3965584e-05	0.016
CH2	0.0074684164	3.3965584e-05	0.008
CH2	0.0074684164	3.3965584e-05	-0.01
CH2	0.0074684164	3.3965584e-05	-0.001
CH2	0.0074684164	3.3965584e-05	0.013
CH2	0.0074684164	3.3965584e-05	-0.005
CH2	0.0074684164	3.3965584e-05	-0.013
CH2	0.0074684164	3.3965584e-05	0.016
CH2	0.0074684164	3.3965584e-05	0.013
CH3	0.0096138025	2.6646244e-05	-0.018
OM	0.0022619536	7.4149321e-07	-0.648
OM	0.0022619536	7.4149321e-07	-0.648
